# Non-respondents do not bias outcome assessment after cervical spine surgery: a multicenter observational study from the Norwegian registry for spine surgery (NORspine)

**DOI:** 10.1007/s00701-022-05453-x

**Published:** 2022-12-21

**Authors:** Tor Ingebrigtsen, Grethe Aune, Martine Eriksen Karlsen, Sasha Gulati, Frode Kolstad, Øystein P. Nygaard, Anette Moltu Thyrhaug, Tore K. Solberg

**Affiliations:** 1 Department of Clinical Medicine, UiT The Arctic University of Norway, Postbox 6050 Langnes, 9037 Tromsø, Norway; 2 Department of Neurosurgery and Norwegian Registry for Spine Surgery (NORspine), University Hospital of North Norway, Tromsø, Norway; 3 Department of Neurosurgery and National Advisory Unit on Spinal Surgery, St. Olav’s University Hospital, Trondheim, Norway; 4 Department of Neuromedicine and Movement Science, Norwegian University of Science and Technology, Trondheim, Norway; 5 Department of Neurosurgery, Oslo University Hospital, Oslo, Norway

**Keywords:** Healthcare quality improvement, Performance measures, Quality measurement, Surgery

## Abstract

**Background:**

The Norwegian registry for spine surgery (NORspine) is a national clinical quality registry which has recorded more than 10,000 operations for degenerative conditions of the cervical spine since 2012. Registries are large observational cohorts, at risk for attrition bias. We therefore aimed to examine whether clinical outcomes differed between respondents and non-respondents to standardized questionnaire-based 12-month follow-up.

**Methods:**

All eight public and private providers of cervical spine surgery in Norway report to NORspine. We included 334 consecutive patients who were registered with surgical treatment of degenerative conditions in the cervical spine in 2018 and did a retrospective analysis of prospectively collected register data and data on non-respondents’ outcomes collected by telephone interviews. The primary outcome measure was patient-reported change in arm pain assessed with the numeric rating scale (NRS). Secondary outcome measures were change in neck pain assessed with the NRS, change in health-related quality of life assessed with EuroQol 5 Dimensions (EQ-5D), and patients’ perceived benefit of the operation assessed by the Global Perceived Effect (GPE) scale.

**Results:**

At baseline, there were few and small differences between the 238 (71.3%) respondents and the 96 (28.7%) non-respondents. We reached 76 (79.2%) non-respondents by telephone, and 63 (65.6%) consented to an interview. There was no statistically significant difference between groups in change in NRS score for arm pain (3.26 (95% CI 2.84 to 3.69) points for respondents and 2.77 (1.92 to 3.63) points for telephone interviewees) or any of the secondary outcome measures.

**Conclusions:**

The results indicate that patients lost to follow-up were missing at random. Analyses of outcomes based on data from respondents can be considered representative for the complete register cohort, if patient characteristics associated with attrition are controlled for.

## Introduction

The purpose of national clinical-quality registries is to monitor, benchmark, and improve the quality of care. Registry data can be used to determine whether patients have timely access, whether care is in accordance with evidence-based guidelines, and to drive improvement in routine clinical practice. Registries are particularly appropriate where there is variation in provider performance and clinical practice and when provider-specific outcomes are benchmarked and compared transparently [16]. Data from registries that produce patient-specific predictors of outcomes can be utilized to develop decision support tools and thus facilitate shared decision-making between patients and providers [9, 29]. Integration between clinical-quality registries and electronic health records entails a potential to leverage the complete experience from all previously registered cases to advise decisions about subsequent patients.

Methodologically, registries are large observational cohorts and as such at risk for attrition bias. The Norwegian registry for spine surgery (NORspine) was established in 2006 to record operations for degenerative disease of the lumbar spine. Cervical spine operations have been recorded since 2012 [24].

It is commonly assumed that a response rate above 80% is necessary to achieve a representative sample of outcomes in clinical trials and cohort studies, because differences in baseline characteristics between respondents and non-respondents can indicate that attrition bias may exist [1, 13, 27]. For national clinical-quality registries assessing all patients treated in routine clinical practice, it is too resource-demanding and expensive to vigorously trace and retain all cohort members [8, 20]. This means the proportion lost to follow-up will be higher than in a limited clinical trial.

The potential attrition bias caused by loss to follow-up has not been evaluated previously for the NORspine cohort of patients operated for degenerative conditions of the cervical spine. Therefore, we aimed at examining whether clinical outcomes differ between respondents and non-respondents in this register cohort.

## Methods

### Study design

This is a retrospective analysis of prospectively collected data comparing respondents and non-respondents to the standardized 12-month follow-up in the NORspine cohort for surgical treatment of degenerative conditions of the cervical spine. We collected outcome data on non-respondents by telephone interview and compared their baseline characteristics and outcomes with those of the respondents. The results are reported according to the Strengthening the Reporting of Observational Studies in Epidemiology (STROBE) guidelines [28].

### Patient and public involvement

The Norwegian association for patients with spine disorders (Ryggforeningen) is represented on NORspine’s advisory board, which approved data extradition to this study. Patients or the public were otherwise not involved in the design, conduct, reporting, or dissemination plans of our research.

### Setting

The NORspine cohort for cervical operations was established in 2012 for quality assessment and research. Participation in NORspine is not mandatory for patients, and it is not required for access to health care or for providers to be eligible for payment. The registry has now (2022) recorded more than 10,000 operations, and approximately 1200 new cases are added each year. All operations in Norway are performed by specialists or residents in training in neurosurgery.

All patients who undergo surgery for degenerative conditions of the cervical spine are eligible to participate. Exceptions are those precluded from consenting because of cognitive failure, a severe psychiatric disorder, language barriers, and children under the age of 16 years. Patients operated due to fracture, primary infection, or tumor of the spine are not eligible.

NORspine routinely calculates its inclusion rate every second year. In 2017, the national coverage for cervical operations was 100% at the institutional level, i.e., all eight providers (five public and three private hospitals) reported. The coverage rate at the individual level was 78% [24].

NORspine collects data from surgeons and patients. Surgeons report immediately after the operation on the diagnosis, comorbidities, and surgical details. At admission for surgery (baseline), patients complete self-administered questionnaires and report about demographics, lifestyle-related factors, and outcome and experience measures (PROMs and PREMs). Follow-up questionnaires are distributed by post directly to patients by the NORspine office at 3- and 12-month follow-ups. Non-respondents receive one postal reminder. Patients respond without the involvement of the treating surgeon or hospital.

### Participants and study size

This study analyzed consecutive patients recorded between February 6 and May 22, 2018. Power analysis indicated that group sizes of at least 21 cases were required to detect a between-group difference of 2.5 points in the arm and neck pain on the numeric rating scale (NRS) with a standard deviation (SD) of 2.9 points and 80% power (*P* value < 0.05) [3, 19]. Because the SD was retrieved from a larger NORspine population, we chose to increase the sample size three-fold, to at least 63 patients in each group. The study period was chosen to define the most recent sample with sufficient size, available for analysis of 12-month outcome data in November 2019.

**Fig. 1:**
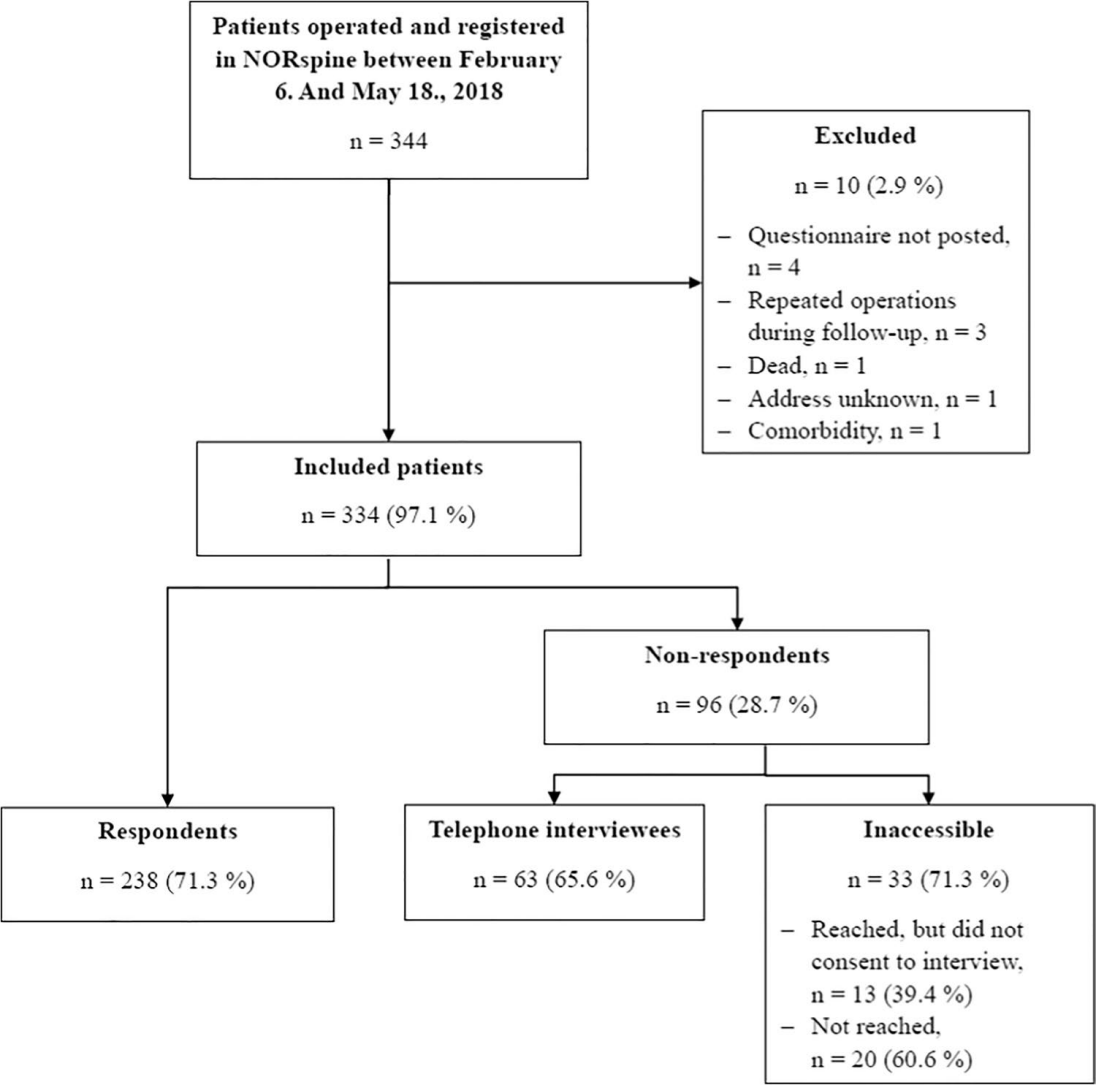
Study flowchart. NORspine, the Norwegian registry for spine surgery

Figure 1 shows the study flowchart. A total of 344 patients were operated and registered in NORspine during the study period and thus eligible. We excluded 10 (2.9%) for reasons listed in the figure and included 334 (97.1%) patients. Among these, 238 (71.3%) responded, and 96 (28.7%) did not respond to the standardized 12-month follow-up.

### Data sources and variables

All data for the respondents were retrieved from NORspine. For the non-respondents, we retrieved data on baseline characteristics from the register, while outcome data were collected by telephone interviews. The telephone interviews were conducted between May 1 and October 31, 2019, implying a mean delay of the 12-month follow-up by 3 months. Table 1 lists the baseline characteristic variables.

**Table 1:** Baseline characteristics of the study participants

Characteristic	Missing, *n* (%)	All (*n* = 334)	Respondents (*n* = 238)	Non-respondents (*n* = 96)		*p* value*	*p* value**
				Telephone interviewees (*n* = 63)	Inaccessible for follow-up (*n* = 33)		
Age (years), mean (95% CI)	0	51.1 (50.0 to 52.2)	52.4 (51.1 to 53.4)	48.1 (45.7 to 50.6)	47.2 (43.5 to 51.0)	0.004	0.008
Sex, female, *n* (%)	0	149 (44.6)	113 (47.5)	24 (38.1)	12 (36.4)	0.184	0.230
EQ-5D-3L score, mean (95% CI)	17 (5.1)	0.47 (0.4 to 0.5)	0.48 (0.4 to 0.5)	0,45 (0.4 to 0.5)	0.39 (0.3 to 0.5)	0.412	0.131
BMI (kg/m^2^), mean (95% CI)	7 (2.1)	27.2 (26.7 to 27.6)	27.0 (26.5 to 27.5)	27.6 (26.6 to 28.7)	27.2 (25.5 to 29.0)	0.280	0.770
LOS (days), mean (95% CI)	0	1.7 (1.5 to 1.9)	1.6 (1.4 to 1.8)	1.8 (1.2 to 2.4)	1.6 (1.0 to 2.3)	0.385	0.951
NRS score arm pain, mean (95% CI)	14 (4.2)	6.1 (5.8 to 6.4)	6.1 (5.8 to 6.4)	6.1 (5.5 to 6.8)	6.2 (5.0 to 7.3)	0.936	0.915
NRS score neck pain, mean (95% CI)	18 (5.4)	6.0 (5.7 to 6.2)	5.9 (5.5 to 6.2)	5.9 (5.3 to 6.6)	6.7 (6.0 to 7.7)	0.837	0.048
Living alone, *n* (%)	4 (1.2)	75 (22.5)	50 (21.0)	17 (27)	8 (24.2)	0.774	0.723
Native Norwegian speaker, *n* (%)	0	305 (91.3)	224 (94.1)	55 (87.3)	26 (78.8)	0.065	0.002
Level of education^a^, *n* (%)	10 (3.0)					0.910	0.626
Current smoker, *n* (%)	3 (0.9)	98 (29.3)	62 (26.1)	25 (39.7)	11 (33.3)	0.084	0.602
Receiving sick-leave benefit, *n* (%)	4 (1.2)	179 (54.2)	120 (51.1)	40 (64.5)	19 (57.6)	0.059	0.483
ASA score^b^, *n* (%)	0					0.257	0.455
Duration of neck pain > 1 year, *n* (%)	11 (3.3)	167 (51.7)	109 (48.0)	38 (60.3)	20 (60.6)	0.084	0.177
Duration of arm pain > 1 year, *n* (%)	8 (2.4)	144 (44.2)	97 (42.2)	32 (50.8)	15 (45.5)	0.222	0.722
Anxiety and/or depression, *n* (%)	7 (2.1)	114 (34.1)	77 (33.0)	23 (37.1)	14 (43.8)	0.549	0.232
Previous cervical spine surgery, *n* (%)	0	54 (16.2)	32 (13.4)	17 (27.0)	5 (15.2)	0.010	0.789
Myelopathy, *n* (%)	0	47 (14.1)	36 (15.1)	8 (12.7)	3 (9.1)	0.628	0.355
Type of operation^c^, *n* (%)	0					0.147	0.873

^*^p values for comparisons between respondents and telephone interviewees

^**^p values for comparison between respondents and patients inaccessible for follow-up

^a^Level of education categorized as primary school, secondary school (vocational), secondary school (grammar), college or university < 4 years, and college or university ≥ 4 years

^b^ASA score categorized as Grade I, II, or III

^c^Type of operation categorized as anterior discectomy and/or decompression with fusion, posterior decompression, or other

*CI*, confidence interval; *EQ-5D-3L*, EuroQol 5-dimensions 3 levels; *BMI*, body mass index; *LOS*, length of hospital stays; *NRS*, numeric rating scale; *ASA*, American Society of Anesthesiologists classification of physical health

The primary outcome measure was patient-reported change in arm pain, and the secondary outcome measures were patient-reported change in neck pain, change in health-related quality of life, and the perceived benefit of the operation.

Patients assessed arm and neck pain intensity with the NRS, ranging from no (0) to the worst conceivable (10) pain [7, 10].

Health-related quality of life was assessed with EuroQol 5 Dimensions 3 Levels (EQ-5D-3L) [6]. EQ-5D is a generic measure which is valid, reliable, and responsive across different conditions and populations. The questionnaire comprises five dimensions, describing different aspects of health, with questions concerning mobility, self-care, usual activities, pain/discomfort, and anxiety/depression. The index value set has been validated in a previous Norwegian cohort [22]. Health state index scores range from − 0.594 to 1, where 1 corresponds to perfect health and 0 to death.

Patients assessed the benefit of the operation by the Global Perceived Effect (GPE) scale, which is a balanced 7-point Likert scale [12]. They answered the question: “To what degree did you benefit from the operation?” The grades range from “completely recovered” to “worse than ever.” The test–retest reliability is excellent in patients with musculoskeletal disorders [12].

We identified the 96 patients who did not respond to the standardized 12-month follow-up after one postal reminder in the register, retrieved their contact information (address and mobile phone number), and used a standardized procedure to contact them: authors GA or MEK primarily called the mobile phone number they had provided. When this was unsuccessful, we secondarily used phone numbers registered in the electronic health record and/or publicly available telephone directories. We first called the phone number(s) twice. If contact was not achieved, we sent a text message with information about the study, and then attempted to call iteratively another three times.

Patients who answered received oral information about the study and were invited to consent to participation. We categorized the patient as inaccessible for the study in cases where contact was not achieved or if consent to participation was not granted.

We verified participants’ identity by asking them to provide their unique national personal identification number and collected their outcome data through a structured interview. The interview guide comprised questions about the 13 outcome measures we considered most relevant and feasible to collect by telephone, among the 37 measures collected with the standardized 12-month follow-up questionnaire, i.e., working status, use of any painkillers, the presence of arm and neck pain, assessment of health-related quality of life, and assessment of the benefit of the operation. All questions were formulated and presented sequentially according to the standardized postal questionnaire. In addition, we asked an open question about reasons for not responding to the questionnaire. The answers were interpreted by the interviewer and the causes categorized as “forgetfulness,” “questionnaire fatigue,” “disability not related to the cervical condition,” “bustle,” “felt so healthy that there was no point in responding,” “discouraged because of an unfavourable outcome of the operation,” “did not receive the questionnaire,” or “other”. We also asked an open question about possible improvements to increase the response rate.

### Statistical analysis

We used the Statistical Package for the Social Sciences (SPSS; version 26 (IBM Armonk, NY: IBM Corp)). All continuous data were normally distributed (Kolmogorov–Smirnov test). We present descriptive data as means with 95% confidence intervals (CI) for continuous variables and counts with percentages for proportions. Differences between groups were examined with one-way analysis of variance (ANOVA) for continuous and Pearson’s chi-squared test for categorical variables. The level of significance was set < 0.05.

We used multivariable binary logistic regression to identify predictors for not responding to the standardized 12-month follow-up. First, possible predictors were identified in univariable analysis (Table 1). Variables associated with not responding (*p* < 0.1) were checked for interactions and collinearity, and then included in multivariable analysis to identify independent predictors (*p* < 0.05).

Table 1 shows that the completeness of data was high, with missing data at baseline for between 0 and 5.4% of the patients for the variables analyzed. The proportion with missing data for the primary outcome measure was 4.2%. We included all cases in the analyses, irrespectively of missing data.

### Ethical considerations

All participants had signed a written consent and granted permission to collect, retain, and use their information for research and quality improvement prior to registration in NORspine. The consent included a permission to being contacted in writing or by telephone in addition to the standardized follow-ups. The data protection officer at the University Hospital of North Norway defined the study as quality improvement and granted approval (file number 02285).

## Results

### Participants

The flowchart in Fig. 1 shows that we included 334 patients (149 (44.6%) woman and 185 (55.4%) men) with a mean age of 51.1 (95% CI 50.0 to 52.2) years. We reached 76 (79.2%) of the 96 non-respondents by telephone and 63 (65.6%) consented to provide outcome data by telephone interview. Thirty-three patients (34.3% of the non-respondents and 9.9% of the total study population) were inaccessible. Of those, 13 (39.4%) declined the interview and 20 (60.6%) could not be reached at all.

Table 1 shows participants’ baseline characteristics, with the non-respondents sub-categorized as telephone interviewees and inaccessible patients. There were few differences between the groups. The telephone interviewees and the inaccessible patients’ mean age was 48.1 (95% CI 45.7 to 50.6) and 47.2 (95% CI 43.5 to 51.0) years, respectively. They were younger than the respondents, who had a mean age of 52.4 (51.1 to 53.4) years. The inaccessible patients had more preoperative neck pain (NRS score 6.7 (95% CI 6.0 to 7.7)) than the respondents (NRS score 5.9 (95% CI 5.5 to 6.2)), and the proportion of native Norwegian speakers (78.8%) was lower among inaccessible patients than respondents (94.1%) (*p* = 0.002). The proportion who had undergone previous cervical spine surgery (27.0%) was higher among telephone interviewees than respondents (13.4%) (*p* = 0.010). There were no significant between-group differences in the proportion with myelopathy.

### Primary analysis

Table 2 shows the between-group comparison of primary and secondary outcome measures for respondents to the standardized postal 12-month follow-up versus the telephone interviewees. There was no statistically significant difference in change in NRS score for arm pain, which was 3.26 (95% CI 2.84 to 3.69) points for the respondents and 2.77 (1.92 to 3.63) points for the telephone interviewees, and no statistically significant differences between the groups for any of the secondary outcome measures.

**Table 2:** Outcomes 12 months after surgical treatment of degenerative conditions of the cervical spine

Outcome	Respondents (*n* = 238)	Telephone interviewees (*n* = 63)	*p* value
NRS score for arm pain, mean change (95% CI)	3.26 (2.84 to 3.69) Missing *n* = 15	2.77 (1.92 to 3.63) Missing *n* = 1	0.299
NRS score for neck pain, mean change (95% CI)	2.57 (2.18 to 2.96) Missing *n* = 6	2.44 (1.78 to 3.11) Missing *n* = 2	0.755
EQ-5D-3L score, mean change (95% CI)	0.22 (0.17 to 0.27) Missing *n* = 18	0.20 (0.12 to 0.28) Missing *n* = 4	0.746
GPE score, grades 5–7, *n* (%)	196 (82.4) Missing *n* = 0	48 (77.3) Missing *n* = 1	0.375

*NRS*, numeric rating scale; *CI*, confidence interval; *EQ-5D-3L*, EuroQol 5-dimensions 3 levels; *GPE*, global perceived effect scale

### Secondary analyses

Table 3 shows results from analyses of predictors for not responding. In univariable analyses, age, non-native Norwegian speaker, receiving sick leave benefit, duration of neck pain of more than 1 year, and having undergone previous cervical spine surgery were associated with not responding. In multivariable analysis, only younger age (OR 0.96, 95% CI 0.93 to 0.98), non-native Norwegian speaker (OR 2.88, 95% CI 1.26 to 6.56), and having undergone previous cervical spine surgery (OR 2.20, 95% CI 1.16 to 4.17) were statistically significant independent predictors for not responding.

**Table 3:** Results of multivariate logistic regression analysis of predictors for not responding to the standardized 12-month questionnaire-based follow-up after cervical spine operations

Variable	Univariate analysis			Multivariate analysis		
	OR	95% CI	*p*-value	OR	95% CI	*p*-value
Older age (years)	0.96	(0.93 to 0.98)	< 0.001	0.96	(0.93 to 0.98)	0.002
Non-native Norwegian speaker (yes/no)	2.96	(1.37 to 6.41)	0.006	2.88	(1.26 to 6.56)	0.012
Receiving sick leave benefit (yes/no)	1.57	(0.97 to 2.56)	0.069	1.31	(0.78 to 2.19)	0.308
Preoperative duration of neck pain > 1 year (yes/no)	1.65	(1.02 to 2.68)	0.042	1.48	(0.89 to 2.47)	0.135
Previous cervical spine surgery (yes/no)	1.91	(1.05 to 3.50)	0.035	2.20	(1.16 to 4.17)	0.015

When the telephone interviewees were invited to explain why they had not responded to the postal questionnaire, 24/63 (38.7%) mentioned various causes other than those in the pre-specified categories, most commonly problems with choosing the correct alternative when answering questions collecting category data (12/63, 19.0%). Forgetfulness (21/63, 33.3%), bustle (9/63, 14,3%), did not receive the questionnaire (3/63, 4.8%), and disability not related to the cervical condition (3/63, 4.8%) were the most frequent pre-specified causes.

The question about possible measures to increase the response rate was answered by 33/63 (52.0%) telephone interviewees. The most frequent recommendations were electronic distribution of the questionnaire (16/33, 48.5%), routine use of telephone interview instead of postal questionnaires (9/33, 27.3%), and simplification of the questionnaire (6/33, 18.2%).

## Discussion

### Key results

We studied a sample from the NORspine-cohort for surgical treatment of degenerative conditions of the cervical spine and found no differences in patient-reported outcomes between respondents to the standardized 12-month follow-up and non-respondents who consented to a telephone interview. The non-respondents were younger and had slightly more neck pain at baseline. Larger proportions were non-native Norwegian speakers, indicating they were immigrants, or had undergone previous cervical spine surgery, compared to respondents. The telephone interviewees gave various explanations for not responding. Their most frequently recommended intervention to increase the response rate was to provide an option for electronic reporting.

### Relation to other studies

Numerous studies have reported differences in baseline characteristics between patients captured and those lost to follow-up in clinical registries, and there is concern that this can indicate that attrition bias may exist, especially if the proportion lost to follow-up exceeds 20% [1, 13]. Typically, non-respondents in spine registries are younger and have a less favorable health status at baseline, and the proportion of males, smokers, and participants with low socioeconomic status is higher than for respondents [2, 5, 11, 14, 17, 18]. These differences in baseline characteristics correspond to those observed in the present study.

Our main finding nevertheless suggests that the response rate of 71.3% was sufficient to achieve estimates of patient-reported outcomes that are representative for the complete cervical spine cohort in NORspine. We are not aware of comparable population-based multicenter studies from clinical-quality registries for cervical spine surgery.

The finding is in line with three previous Scandinavian studies that collected 12-month outcome data from non-respondents to national clinical-quality registries for lumbar spine surgery [5, 11, 23]. In a previous study, we interviewed 142 (22%) non-respondents in a single-center sample of 633 patients who were registered in a precursor of NORspine. We found no statistically significant differences in outcomes between respondents and non-respondents [23]. Højmark and coworkers also used a structured telephone interview and collected outcome data from non-respondents to the Danish national spine database (DaneSpine) [11]. They managed to interview all 57 (12%) non-respondents among 473 patients treated at a single center and found no differences in outcomes. Elkan and coworkers compared non-respondents (27%) in a cohort (*n* = 7791) from the equivalent Swedish registry (Swespine) with data from a single-center prospective study (*n* = 177) which included similar patients and had only 2% non-respondents [5]. There were no statistically significant differences in outcomes.

Other previous studies of participants lost to follow-up after lumbar spine surgery show conflicting results. A study of 289 patients who underwent minimally invasive surgery at a single center collected 12-month outcome data from 53 (53.5%) of 99 non-respondents (34% of the total sample) through e-mailed surveys or telephone interviews and found that patients lost to follow-up reported greater improvement than those who continued to follow-up [4]. A study of 316 patients operated by a single surgeon with lumbar fusion compared 76 (24%) patients who completed a program of multiple questionnaire-based follow-ups over 2 years with 240 (76%) who responded incompletely [18]. In this study, outcomes were less favorable for patients with incomplete follow-ups. A recent study merged data from a large cohort in Swespine (*n* = 18,911) and other public registries, used regression analysis to identify variables associated with not responding, and predicted non-respondents’ outcomes [17]. These were significantly worse than those reported by respondents, but the differences in the proportion reaching a successful outcome were not large (lumbar spinal stenosis 3.3, lumbar disc herniation 4.3, and degenerative disc disorder 4.8 percentage points).

### Interpretation

In the present study, attrition of almost 30% was not associated with differences in outcomes between respondents and non-respondents. However, careful evaluation of the causes for attrition is probably more important than the emphasis on the proportion only, as patients lost to follow-up could be missing completely at random (MCAR), at random (MAR), or not at random (MNAR) [13].

In MCAR observations, the respondent and non-respondent groups are similar at baseline and report the same clinical outcome, meaning that the non-respondents are a genuine random sample of the original cohort. This is however unlikely, because patients lost to follow-up often have certain characteristics. In MAR observations, loss to follow-up may be associated with certain patient characteristics, but not with the outcome. In this situation, bias can be reduced by controlling for the covariates that are associated with attrition. In MNAR observations, the probability of loss to follow-up is associated with the outcome. This means that outcome assessment for the complete cohort based only on the respondents will be biased [13]. Such bias is a recognized challenge in studies based on data from spine registries [27]. Strategies to manage MNAR observations include multiple imputations and the use of mixed linear models [26].

In the present study, non-respondence was associated with lower age, being a non-native Norwegian speaker, and having undergone previous cervical spine surgery. The absence of differences in outcomes between respondents and telephone interviewees indicates that non-respondents were MAR. This implies that analyses of outcomes based on data from respondents can be considered representative for the complete NORspine cohort, if baseline variables associated with not-responding are adequately controlled for.

Regardless, measures to increase the response rate should be undertaken. NORspine has recently translated its questionnaires to English to support participation from immigrants, and an electronic system for follow-up has been implemented. These measures are supported by the risk factors for not responding and the recommendations from non-respondents identified in the present study.

### Limitations

There were some differences between the study groups at baseline, e.g., a higher proportion who had undergone previous cervical spine surgery among the telephone interviewees than among the respondents. We cannot rule out that this could cause attrition bias.

It also is a weakness that only 65.6% of the non-respondents could be reached and consented to telephone interviews. The inaccessible patients constituted 9.9% of the complete cohort. They could be MNAR and have outcomes either better or worse than those of respondents and telephone interviewees. However, the differences between telephone interviewees and inaccessible patients at baseline were small, and we therefore consider it unlikely that they had different outcomes. Furthermore, the telephone interviews were done with an average delay of 3 months compared to the standardized 12-month follow-up. This could introduce recall bias. We consider this unimportant, as previous reports have shown that outcomes are stable between 1 and 2 years [25]. Also, telephone interviews could over- or underestimate outcomes, compared to postal questionnaires. However, a meta-analysis compared different modes of administration of PROMs and concluded they were interchangeable [21]. Finally, confounders not measured by NORspine could be associated with loss to follow-up.

The coverage rate for cervical operations in NORspine was 78% at the individual level in 2017. Drop-out analysis based on the comparison of register data with hospital-administrative data showed that patients undergoing acute operations were under-represented [24]. This selection implies a likely underestimation of the mean treatment effect, since such patients experience more improvement than those undergoing scheduled surgery [15]. Accordingly, we do not know whether the NORspine cohort is representative for all patients undergoing cervical spine operations. Furthermore, there are differences in contextual societal factors and the organization of healthcare between Norway and larger non-Scandinavian countries that could limit the external validity of our findings.

The multicenter population-based design is a strength. Also, the telephone interviews were done by medical students with no relation to patients or providers, and they were blinded to the outcomes of the respondents when they interviewed non-respondents.

## Conclusions

We compared respondents and non-respondents to a standardized 12-month follow-up in a national clinical-quality register for cervical spine surgery and found no differences in outcomes. This indicates that patients lost to follow-up were missing at random. The findings thus imply that analyses of outcomes based on data from the respondents can be considered representative for the complete register cohort, if patient characteristics associated with attrition are adequately controlled for.

## Data Availability

Data availability is restricted due to their sensitive nature. Anonymized data are available on reasonable request.
